# Exosome-like nanovesicles from acerola for CRISPR-Cas9 ribonucleoprotein delivery to the central nervous system

**DOI:** 10.1016/j.omtn.2026.102896

**Published:** 2026-03-12

**Authors:** Yui Nagamatsu, Tomohiro Umezu, Taehun Hong, Takahide Niijima, Shin-ichiro Ohno, Yuichiro Harada, Kohsuke Kanekura, Takahiro Ochiya, Masahiko Kuroda

**Affiliations:** 1 Department of Molecular Pathology, Tokyo Medical University, Tokyo, Japan; 2 Department of Pharmacology, Tokyo Medical University, Tokyo, Japan; 3 Department of Molecular and Cellular Medicine, Institute of Medical Science, Tokyo Medical University, Tokyo, Japan

**Keywords:** MT: Delivery Strategies, acerola, blood-brain barrier, central nervous system, CRISPR/Cas9, drug delivery, neurodegenerative disease, plant-derived exosomes

## Abstract

An aberrant six-base repeat in intron 1 of *C9orf72* is the most frequent cause of solitary and familial amyotrophic lateral sclerosis and frontotemporal dementia. This mutation is a potential target for CRISPR/Cas9-based genome editing. However, the blood-brain barrier and limitations of current viral or nanoparticle-based delivery systems to neurons significantly restrict the clinical application of CRISPR-Cas9 in the brain. To address these challenges, we developed a drug delivery system using acerola-derived exosome-like nanoparticles (AELNs), which may overcome several limitations associated with human exosomes. AELNs stably form complexes with ribonucleoproteins (RNPs) comprised of Cas9 proteins and guide RNAs (gRNAs). We improved the delivery efficiency and selectivity of AELN/RNP complexes in GLP2-receptor-expressing neurons by incorporating GLP2 peptides into the AELN/RNP complexes. Intranasal administration of peptide-tagged AELN/RNP complexes *in vivo* confirmed the successful genome editing of *C9orf72*, demonstrating the potential of this system for treating neurodegenerative diseases. This study presents a potentially innovative approach for *in vivo* genome editing using a noninvasive delivery system.

## Teaser

Kuroda and colleagues report that acerola-derived exosome-like nanovesicles efficiently deliver CRISPR-Cas9 into the brain via intranasal administration. This non-invasive strategy enables targeted editing of the ALS-associated C9orf72 gene, highlighting plant-derived vesicles as scalable and safe carriers for future gene therapies in neurodegenerative diseases.

## Introduction

Efficient and safe delivery remains a major barrier to clinical translation of CRISPR/Cas9 genome editing, particularly for neurological disorders.^1^^,^^2^ Conventional vectors, including viruses and synthetic nanoparticles, present safety and efficacy limitations.^3^^,^^4^^,^^5^^,^^6^ Extracellular vesicles (EVs) have emerged as promising carriers because they are biocompatible, protect genetic cargo from degradation,^5^^,^^6^^,^^7^ and can traverse biological barriers such as the blood-brain barrier (BBB).^6^^,^^7^^,^^8^^,^^9^ However, the clinical application of EV-mediated CRISPR/Cas9 delivery is constrained by challenges^5^^,^^6^^,^^9^ in scalable EV production, inefficient cargo loading,^10^ and limited understanding of EV transport mechanisms and immune interactions.^10^^,^^11^^,^^12^

We have developed EV-based drug delivery systems (DDSs), including genetically modified exosomes designed for tissue-specific delivery.^13^^,^^14^ Nevertheless, clinical translation has been hindered by limitations in large-scale purification^15^ and inefficient nucleic acid loading.^16^ To overcome these issues, we investigated exosomes derived from edible plants rather than animal cells. Plant-derived exosomes are resistant to digestive enzymes such as pepsin and bile acids, suggesting their potential as DDSs that protect cargo and enable oral administration.^17^ However, prior studies primarily evaluated functional delivery rather than definitive vesicular encapsulation or physicochemical protection. We identified acerola-derived exosome-like nanoparticles (AELNs) as a promising platform for clinical application. AELNs can be produced at low cost, and in large quantities. They form stable nucleic acid complexes that resist enzymatic degradation and have been shown to reach the brain following oral administration in mice.^17^ These findings suggest that AELNs may be applicable for treating neurological diseases.

Gene therapy has gained attention for the treatment of amyotrophic lateral sclerosis (ALS). Expansion of a noncoding GGGGCC hexanucleotide repeat (HRE) in the Chromosome 9 Open Reading Frame 72 (*C9orf72*) gene is the most common genetic cause of ALS, with repeat numbers reaching into the thousands of affected individuals.^18^ CRISPR/Cas9-mediated removal of this repeat using adeno-associated virus vectors significantly reduces ALS-associated pathology in mouse models.^19^

In this study, we evaluated AELNs as a noninvasive delivery system for CRISPR/Cas9 ribonucleoprotein complexes to neurons. We assessed their ability to transport both nucleic acids and proteins, including Cas9, and enhance delivery efficiency through glucagon-like peptide 2 (GLP2) peptide modification. These findings provide a noninvasive alternative to current delivery strategies and advance gene therapy for neurological diseases such as ALS.

## Results

### Extraction, characterization, and intracellular delivery visualization of AELNs

**Figure 1:**
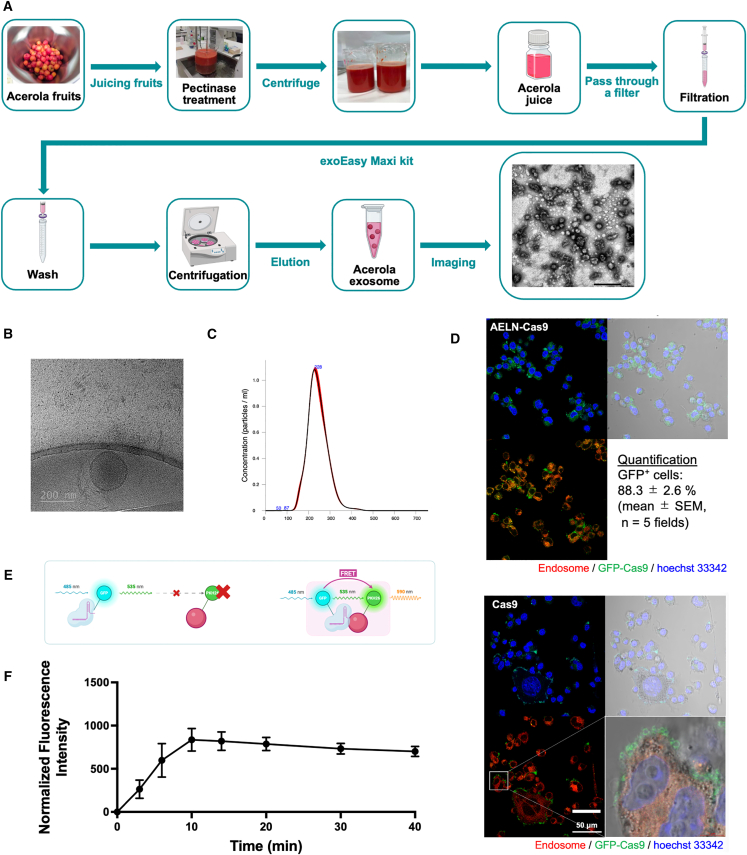
Extraction, characterization, and intracellular delivery of acerola-derived exosome-like nanovesicles (AELNs) (A) Schematic of the isolation process of AELNs from acerola juice using the exoEasy Maxi Kit (Qiagen), followed by ultracentrifugation at 100,000 × g for 70 min at 4°C. Representative transmission electron microscopy (TEM) image of AELNs obtained by negative staining, shown as a qualitative visualization of isolated particles. (B) Cryogenic transmission electron micrograph of the isolated AELNs, prepared on lacey carbon grids and imaged at 200 kV. Scale bars: 200 nm. (C) Nanoparticle tracking analysis performed with a NanoSight LM10 system on an AELN suspensions diluted in PBS. Particle size distribution and concentration data are represented as mean ± SD from *n* = 3 independent isolations, each measured in triplicate. (D) Fluorescence micrographs of HEK293 cells incubated for 4 h with AELNs complexed with GFP-fusion Cas9 ribonucleoproteins (RNPs) or with RNPs alone. AELNs and RNPs were mixed at a 10:1 particle-to-protein molar ratio and incubated at 4°C for 30 min before addition to cells. Green, GFP-Cas9; red, endosomal marker; blue, Hoechst 33342 (nuclei). Phase-contrast and merged images are shown. Scale bars: 50 μm. Representative images from three independent experiments. Quantification of uptake was performed by calculating the percentage of cells showing detectable intracellular GFP signal (GFP^+^ cells/Hoechst^+^ nuclei) from five independent microscopic fields (mean ± SEM). (E) Schematic of Förster Resonance Energy Transfer (FRET) analysis in which PKH26-labeled AELNs were mixed with GFP-fusion Cas9 RNPs and fluorescence emission monitored at 590 nm. (F) FRET analysis of AELN/RNP interactions. Time course fluorescence measurements performed at room temperature for up to 30 min after mixing. Data are presented as mean ± SD from *n* = 3 independent experiments.

AELNs were successfully isolated from freshly squeezed acerola juice and subsequently characterized by electron microscopy and nanoparticle tracking analysis (Figure 1A). Transmission electron microscopy (TEM; Figure 1A) provided an overview of the isolated AELNs, while cryogenic electron microscopy (cryo-EM; Figure 1B) revealed that AELNs were spherical nanoparticles surrounded by a lipid bilayer, with diameters of approximately 200 nm. Nanoparticle tracking analysis (NTA) demonstrated a mean particle diameter of 230 ± 50 nm (range: 170–310 nm) and yield of approximately 4 × 10^10^ particles per 1 mL of acerola juice, highlighting the scalability of AELN production for potential clinical applications (Figure 1C).

The intracellular delivery capability of AELNs was evaluated using fluorescence microscopy. GFP-fusion Cas9 protein and guide RNA (gRNA) complexes, collectively referred to as ribonucleoproteins (RNPs), were combined with AELNs and introduced into HEK293 cells. GFP signals were readily detected within the majority of cells, indicating successful intracellular delivery of the RNPs (Figure 1D). Quantitative analysis showed that 88.3% ± 2.6% of cells exhibited detectable intracellular GFP signal (mean ± SEM, *n* = 5 microscopic fields). In contrast, treatment with RNPs alone showed GFP signals largely confined to the cell surface, with minimal intracellular localization (Figure 1D).

The complex formation capacity of AELNs with RNPs was further confirmed through Förster resonance energy transfer (FRET) analysis. AELNs labeled with PKH26 generated FRET signals at a wavelength of 590 nm only when in proximity to GFP-fusion Cas9 proteins (Figure 1E). Time course monitoring revealed that the FRET fluorescence intensity increased within 5 min of mixing, peaked at 10 min, and remained stable for up to 40 min post-mixing (Figure 1F). These findings suggest that AELNs rapidly form stable complexes with RNPs, achieving maximal complex formation within 10 min.

### Efficiency of AELNs as carriers for CRISPR/Cas9 RNP complexes

**Figure 2:**
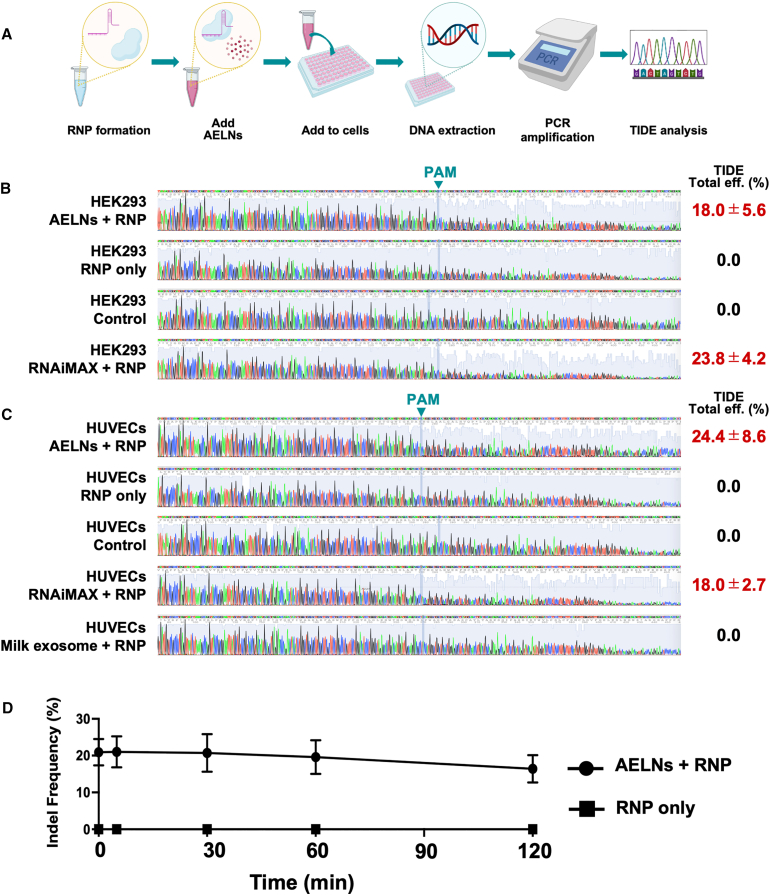
Efficient genome editing in cultured cells using acerola-derived exosome-like nanovesicles (AELNs) as carriers for CRISPR/Cas9 ribonucleoprotein (RNP) complexes (A) Schematic representation of the experimental workflow for genome editing in cultured cells using AELN-mediated delivery of CRISPR/Cas9 RNP complexes. AELNs were mixed with RNPs, incubated on ice for 20 min, and applied to cells. (B) Representative Sanger sequencing chromatograms and Tracking of InDels by Decomposition (TIDE) analysis of genomic DNA from HEK293 cells following AELN-mediated RNP delivery, compared with RNAiMAX transfection. Data are presented as mean ± SD from *n* = 3 independent experiments. (C) Representative Sanger sequencing chromatograms and TIDE analysis of genomic DNA from human umbilical vein endothelial cells (HUVECs) following AELN-mediated RNP delivery, compared with RNAiMAX and milk-derived exosome carriers. Data are presented as mean ± SD from *n* = 3 independent experiments. (D) Time course serum stability assay assessing the functional stability of AELN/RNP complexes. AELN/RNP complexes (particle-to-protein molar ratio of 10:1) or RNPs alone were incubated in 10% human serum at 37°C for the indicated times (0, 5, 30, 60, and 120 min). Following serum incubation, samples were diluted in complete culture medium and applied to HEK293 cells. Genome-editing efficiency was quantified by Tracking of InDels by Decomposition (TIDE) analysis 24 h after cellular treatment. Data are presented as mean ± SD from *n* = 3 independent experiments. Time 0 min indicates no serum incubation.

AELNs efficiently formed complexes with CRISPR/Cas9 RNPs composed of Cas9 protein and gRNA through simple mixing followed by incubation on ice for 20 min. As confirmed by the time course FRET analysis (Figure 1F), fluorescence intensity increased rapidly within 5 min, peaked at 10 min, and remained stable for at least 40 min, indicating the formation of stable AELN/RNP complexes. These stable complexes enabled the evaluation of their potential for targeted gene editing in cultured cells (Figure 2A). Initial assays used gRNA targeting the α-galactosidase (*GLA*) gene to validate the genome-targeting specificity and efficiency of the system.

When AELN/RNP complexes were introduced into various cell lines, HEK293 cells, known for their high transfection efficiency, exhibited sequence waveform disruptions downstream of the protospacer adjacent motif (PAM) sequence when AELNs or the commercial transfection reagent, RNAiMAX, were used as carriers (Figure 2B). Genome-editing efficiency quantified using the Tracking of InDels by Decomposition (TIDE) analysis showed an indel insertion efficiency of 18.0% ± 5.6% with AELNs, comparable to that of 23.8% ± 4.2% achieved with RNAiMAX (Figure 2B).

Similarly, in human umbilical vein endothelial cells (HUVECs), both AELNs and RNAiMAX, when used as carriers, induced sequence-waveform disruption downstream of the PAM sequence. TIDE analysis indicated an indel insertion efficiency of 24.4% ± 8.6% for AELNs, which exceeded that of 18.0% ± 2.7% achieved with RNAiMAX (Figure 2C).

Further experiments were conducted to confirm the feasibility of genome editing in HUVECs using exosomes other than AELNs as carriers. Knockout of the *GLA* gene in HUVECs was attempted using exosomes derived from human breast milk. Notably, sequence-waveform disruption downstream of the PAM sequence was not observed in cells treated with milk-derived exosomes (Figure 2C), demonstrating that AELNs are highly efficient carriers for delivering CRISPR/Cas9 RNP complexes into the tested cell types, thereby inducing robust genome editing in both HEK293 and HUVEC cells.

To further evaluate the functional stability of AELN/RNP complexes under serum conditions, AELN/RNP complexes were pre-incubated in serum prior to cell treatment. When incubated in 10% human serum, AELN/RNP complexes retained substantial genome-editing activity over time, with indel efficiencies remaining largely unchanged after up to 2 h of serum exposure (Figure 2D). In contrast, RNPs without AELNs showed no detectable genome-editing activity at any time point following serum incubation.

A similar trend was observed in mouse serum, in which AELN/RNP complexes maintained genome-editing activity throughout the 0- to 120-min incubation period, whereas RNP-only controls remained inactive (Figure S1). These results indicate that association with AELNs preserves the functional genome-editing capability of CRISPR/Cas9 RNP complexes under both human and mouse serum conditions.

### Targeted CRISPR/Cas9 editing of the C9orf72 gene for ALS applications

**Figure 3:**
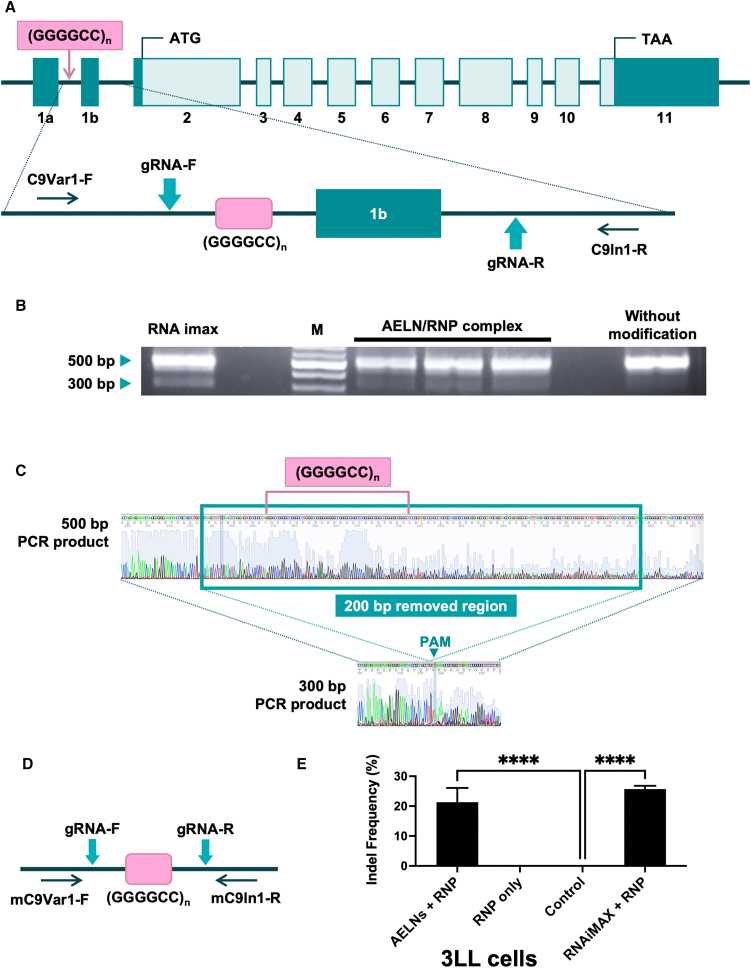
Targeted CRISPR/Cas9 editing of the *C9orf72* gene using acerola-derived exosome-like nanovesicles (AELNs) as carriers (A) Schematic of the human *C9orf72* locus showing the GGGGCC repeat expansion and positions of the two gRNAs designed for excision. (B) PCR analysis of the target region in HEK293 cells treated with AELN-CRISPRCas9 ribonucleoprotein (RNP) complexes. Genomic DNA was amplified using primers flanking the repeat region and PCR products resolved on a 2% agarose gel. (C) Sanger sequencing of the PCR products from (B) confirming excision of approximately 200 bp, including the GGGGCC repeat region. Data are representative of *n* = 3 independent experiments. (D) Schematic of the murine *C9orf72* locus corresponding to the human locus shown in (A). The gRNAs targeting the repeat region are indicated. Data are representative of *n* = 3 independent experiments. (E) Population-level quantification of genome-editing efficiency at the murine C9orf72 locus in 3LL cells. PCR products amplified with primers flanking the repeat region were directly subjected to Sanger sequencing without gel purification, and indel frequencies were quantified by TIDE analysis. Data are presented as mean ± SD from *n* = 3 independent experiments. Statistical analysis was performed using one-way ANOVA followed by post hoc multiple-comparison testing. ∗∗∗∗*p* < 0.0001 versus control.

We compared two gRNAs (gRNA-1 and gRNA-2) designed to flank the CCCCGG repeat region of C9orf72. Both gRNAs showed comparable genome-editing efficiencies in HEK293 cells as quantified by TIDE analysis (20.4% ± 1.2% for gRNA-1 and 19.8% ± 1.8% for gRNA-2; Figure S2). The genome-editing efficiency of a CRISPR/Cas9 system targeting the GGGGCC repeat expansion in the *C9orf72* gene was first evaluated using an *in vitro* model (Figure 3A). In HEK293 cells, the CRISPR/Cas9 system targeting *C9orf72* was delivered using AELNs. PCR amplification of the target region revealed a primary band at approximately 500 bp and an additional band at 300 bp, which is presumed to represent the excision of the GGGGCC repeat region (Figure 3B). Sanger sequencing of these PCR products confirmed that the AELNs/RNP complexes successfully excised approximately 200 bp, including the GGGGCC repeat region, in HEK293 cells (Figure 3C). To quantify editing at the population level, we performed TIDE analysis on the same PCR amplicon without gel purification. TIDE revealed indel frequencies of 22.5% ± 1.8% at the gRNA-1 site and 20.9% ± 2.0% at the gRNA-2 site (Figure S3).

To further assess genome-editing efficiency in a mouse cell line, the CRISPR/Cas9 system was delivered using AELNs as carriers into Lewis lung cancer (3LL) cells, a murine cell line (Figure 3D). Following delivery of the CRISPR/Cas9 system targeting the mouse *C9orf72* gene into 3LL cells, genomic DNA was extracted and analyzed using Sanger sequencing followed by TIDE analysis. Quantitative TIDE decomposition revealed an indel frequency of 21.3% ± 4.8% in AELN/RNP-treated cells, which was comparable to that achieved using RNAiMAX-mediated delivery (25.7% ± 1.1%) (Figure 3E). These results indicate that the AELN/RNP complex effectively facilitates genome editing in mouse cells. Detailed Sanger chromatograms obtained from both sequencing primer directions, together with confidence traces and corresponding TIDE decomposition profiles, are provided in Figure S4.

### Enhancement of delivery efficiency through GLP2 peptide modification

**Figure 4:**
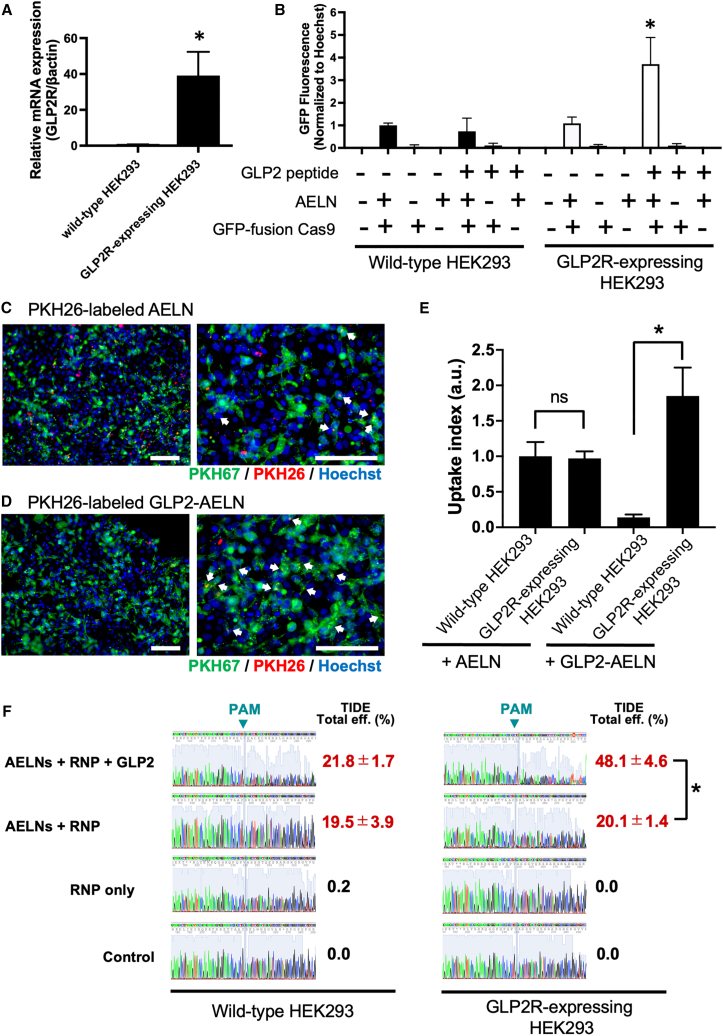
Enhancement of delivery efficiency through GLP2 peptide modification of acerola-derived exosome-like nanovesicles (AELNs) (A) Reverse transcription PCR analysis of glucagon-like peptide 2 receptor (GLP2R) expression in wild-type (WT) and GLP2R-overexpressing (GLP2R+) HEK293 cells. β-actin was used as an internal control. Error bars represent the mean ± SD; ∗*p* < 0.05. (B) GLP2R+ and WT HEK293 cells were seeded in a 96-well plate and treated with GLP2-modified AELN/fluorescently labeled ribonucleoprotein (RNP) complexes. After 24 h of incubation, GFP fluorescence intensity was measured and normalized to the cell count using Hoechst staining to quantify uptake efficiency. Data are presented as mean ± SD (*n* = 3 independent experiments; ≥3 wells per condition); ∗*p* < 0.05. (C) Competitive uptake in mixed cultures of GLP2R+ (pre-labeled with PKH67, green) and WT HEK293 cells. PKH26-labeled GLP2 peptide-modified AELNs (GLP2–AELNs, red) preferentially accumulated in GLP2R + cells within the same field. Representative white arrows indicate PKH26-positive puncta inside PKH67-positive cells. Nuclei were counterstained with Hoechst 33342 (blue). Images are representative of *n* = 3 independent experiments. Scale bars: 500 μm. (D) Competitive uptake of PKH26-labeled unmodified AELNs (red) in GLP2R+ (PKH67+, green) and WT HEK293 cells, showing comparable uptake in both cell types. White arrows highlight representative PKH26-positive puncta. Nuclei were counterstained with Hoechst 33342 (blue). Images are representative of *n* = 3 independent experiments. Scale bars: 500 μm. (E) Quantification of AELN uptake based on mean PKH26 fluorescence intensity per cell in wild-type (WT) and GLP2R-expressing (GLP2R^+^) HEK293 cells co-cultured at a 1:1 ratio and treated with unmodified or GLP2-modified AELNs. Data were normalized to the mean PKH26 intensity of WT cells treated with unmodified AELNs and are presented as mean ± SD (*n* = 3 independent experiments; ≥50 cells per condition). Statistical comparisons between GLP2R^+^ and WT cells were performed within each condition using a paired two-tailed *t* test. ∗*p* < 0.01, ns, not significant. High-resolution images and single-channel views are provided in Figure S4. (F) Sanger sequencing chromatograms and corresponding indel frequencies at the C9orf72 target locus. Representative chromatograms are shown for wild-type and GLP2R-overexpressing HEK293 cells treated with GLP2-modified or unmodified AELN/RNP complexes. Indel frequencies (%) shown adjacent to each chromatogram were quantified by Tracking of InDels by Decomposition (TIDE) analysis of the full mixed Sanger traces. Differences between conditions are primarily reflected in the confidence trace (gray background) and downstream signal heterogeneity, indicating the presence of mixed alleles, rather than in the dominant colored peaks. Data are presented as mean ± SD (*n* = 3 independent experiments). Statistical significance (*p* < 0.05) was determined by comparing GLP2-modified and unmodified AELN/RNP treatments in GLP2R-overexpressing HEK293 cells. PAM, protospacer adjacent motif.

To enhance the effectiveness of AELNs as delivery carriers, GLP2 peptides were incorporated into AELN/RNP complexes to confer specificity toward target cells. This modification aimed to improve the delivery efficiency for GLP2 receptor (GLP2R)-expressing cells, including neurons, with potential applications in ALS-related therapies. Initial reverse transcription PCR (RT-PCR) analysis revealed minimal GLP2R expression in wild-type HEK293 cells. Therefore, GLP2R-overexpressing HEK293 cells were generated. RT-PCR confirmed robust expression of GLP2Rs in these modified cells (Figure 4A).

GLP2-modified AELNs/fluorescently labeled RNP (GFP-fusion Cas9) complexes were introduced into wild-type and GLP2R-overexpressing HEK293 cells, and uptake efficiency was quantified using a fluorescence plate reader. GFP fluorescence intensity, normalized to Hoechst staining for cell count, was significantly increased only in GLP2R-overexpressing HEK293 cells treated with GLP2-modified AELNs, whereas unmodified AELNs showed no significant difference between the two cell types (Figure 4B). This indicates that GLP2 modification enhances selective delivery to GLP2R-expressing cells.

In a competitive co-culture assay, wild-type and GLP2R-overexpressing HEK293 cells (pre-labeled with PKH67) were mixed at a 1:1 ratio. PKH26-labeled AELNs or GLP2–AELNs were then added to the culture. As shown in Figure 4C, unmodified AELNs were taken up to a similar extent by both PKH67-negative (wild-type) and PKH67-positive (GLP2R-overexpressing) cells. In contrast, GLP2-AELNs showed preferential accumulation in PKH67-positive cells (Figure 4D). Because PKH26 and PKH67 signals overlap to yield yellow puncta, the merged images can reduce the visual contrast of colocalized vesicles; therefore, representative puncta are indicated in Figures 4C and 4D, and channel-separated images are provided in Figure S5. Quantitative analysis (Figure 4E) further demonstrated that GLP2-modified AELNs exhibited significantly higher uptake in GLP2R-overexpressing cells compared with wild-type cells, whereas unmodified AELNs showed no significant difference in uptake between the two cell types.

To further evaluate genome-editing efficiency, we compared the indel insertion efficiency between GLP2-modified and unmodified AELN/RNP complexes. In GLP2R-overexpressing HEK293 cells, the indel insertion efficiency was significantly higher with GLP2-modified AELNs (48.1% ± 4.6%) than with unmodified AELNs (20.1% ± 1.4%) (Figure 4F). In contrast, in wild-type HEK293 cells, no significant difference was observed between GLP2-modified and unmodified AELNs, with indel insertion efficiencies of 21.8% ± 1.7% and 19.5% ± 3.9%, respectively (Figure 4F).

These findings highlight the potential of GLP2-modified AELNs as efficient and selective delivery vehicles for RNP complexes, providing a promising approach for targeting GLP2R-expressing cells in therapeutic applications.

### *In vivo* validation of GLP2-modified AELNs in a mouse model

**Figure 5:**
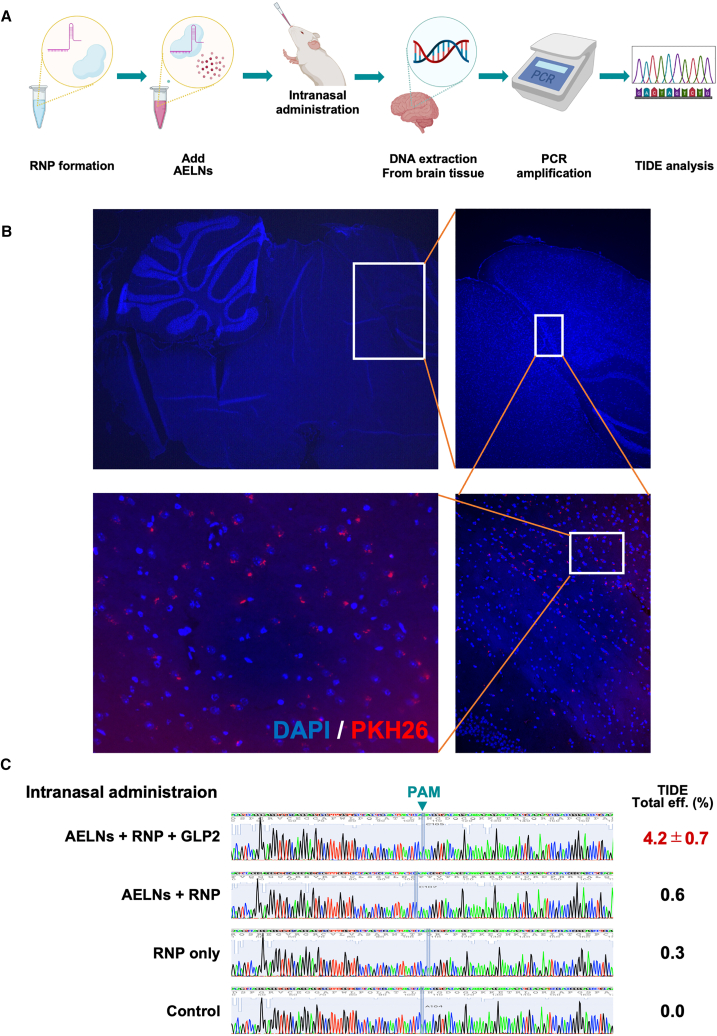
*In vivo* validation of glucagon-like peptide 2 (GLP2)-modified acerola-derived exosome-like nanovesicles (AELNs) in a mouse model (A) Schematic representation of the experimental workflow for *in vivo* genome editing using GLP2-modified AELNs. AELN/ribonucleoprotein (RNP) complexes were administered intranasally, and mouse brain tissues collected 24 h post-administration for genomic analysis. (B) Representative fluorescence images of brain sections collected 24 h after intranasal administration of PKH26-labeled AELNs (red). Nuclei were counterstained with DAPI (blue). Images are shown at progressively higher magnifications, starting from the whole section to the periventricular regions (white boxes indicate zoomed-in areas). PKH26-positive signals were predominantly localized to cell layers lining the ventricular surfaces. Scale bars: 500 μm. Representative images from *n* = 5 mice per group are shown. (C) Tracking of InDels by Decomposition (TIDE) analysis of the *C9orf72* target region in brain tissues following CRISPR/Cas9 delivery. Genomic DNA was extracted from the brains of mice treated with GLP2-modified or unmodified AELN/RNP complexes. Indel insertion efficiency was quantified using TIDE. Data are presented as mean ± SD (*n* = 5 mice per group). PAM, protospacer adjacent motif.

The *in vivo* efficacy of GLP2-modified AELN/RNP complexes was evaluated using a mouse model (Figure 5A). To confirm brain delivery under the same dosing regimen, PKH26-labeled AELNs were administered intranasally to wild-type mice, and brains were collected 24 h later. DAPI-counterstained cryosections revealed PKH26-positive signals predominantly localized to cell layers adjacent to the ventricular surfaces, whereas vehicle-treated controls showed no comparable fluorescence signals (Figure 5B). These findings confirm that AELNs administered intranasally can reach periventricular regions in the brain under our experimental conditions. In contrast, oral administration of PKH26-labeled AELNs using the same dosing regimen did not yield detectable fluorescence signals in brain sections (Figure S6), indicating detectable brain delivery only with intranasal administration in this study.

Next, GLP2-modified AELN/RNP complexes targeting the *C9orf72* gene were administered intranasally to mice, and brain tissues were harvested 24 h later to analyze the target genomic region of the *C9orf72* gene. Using TIDE analysis to quantify genome-editing efficiency, intranasally administered GLP2-modified AELN/RNP complexes achieved an indel insertion efficiency of 4.2% ± 0.7% in brain tissue. In contrast, the GLP2-unmodified AELN/RNP complexes showed no significant indel insertion efficiency (Figure 5C). Genome-editing activity was detectable under our experimental conditions only after intranasal administration, suggesting that the delivery route is crucial for achieving genome editing *in vivo*. Collectively, these findings demonstrate that GLP2-modified AELNs are effective and specific delivery vehicles for CRISPR/Cas9 RNP complexes in the brain.

## Discussion

This study highlights the efficiency and therapeutic potential of AELNs as delivery vehicles for CRISPR/Cas9-based gene editing. We demonstrated the formation of RNPs consisting of Cas9 protein and gRNA complexes with AELNs, achieving precise genome editing in HEK293 cells and HUVECs. Furthermore, GLP2-peptide-modified AELN/RNP complexes successfully targeted the GGGGCC repeat expansions in the *C9orf72* gene in a mouse model, a mutation implicated in ALS. The ability to achieve precise genome editing in brain tissues via noninvasive intranasal administration highlights the potential of AELNs as promising therapeutic tools for neurodegenerative diseases. Furthermore, these findings illustrate the potential of plant-derived exosomes to enable efficient and targeted gene editing *in vitro* and *in vivo*.

The AELN-based CRISPR/Cas9 delivery system offers several advantages over conventional vectors. First, intranasal administration allowed for efficient delivery across the BBB to brain tissues,^20^^,^^21^ avoiding invasive techniques, such as intrathecal injection. This significantly reduces patient burden and improves the feasibility of central-nervous-system-targeted therapies.^22^^,^^23^ Second, the plant-derived nature of AELNs contributes to their biocompatibility, offering reduced immunogenicity compared to animal-derived exosomes or viral vectors.^24^ Additionally, the scalability of AELN production from acerola fruit makes it a cost-effective and practical option for clinical applications.^25^

A major strength of our approach is the direct delivery of RNP complexes, which circumvents the transcription and translation processes required for mRNA- or plasmid-DNA-based approaches,^26^ enhancing genome-editing efficiency and reducing the risk of unintended genetic integration.^27^^,^^28^ The superiority of this approach was further validated using fluorescently labeled Cas9, which visually confirmed the successful intracellular delivery of AELN/RNP complexes. In contrast, naked RNPs failed to penetrate cells, underscoring the critical role of AELNs as delivery carriers.

Moreover, FRET experiments provided insights into the mechanism underlying AELN/RNP complex formation, suggesting that electrostatic interactions facilitate their stabilization,^29^ which likely enhances their uptake by target cells. The addition of GLP2 peptide modifications further improved targeting efficiency. GLP2 may be enriched in the brain owing to its capacity to interact with GLP2Rs, which are reported to be expressed in brain tissues; however, further experimentation is required to confirm this phenomenon.^30^ In this study, we demonstrated that GLP2-modified AELNs preferentially enhanced delivery of RNP complexes to GLP2R-expressing cells, resulting in improved genome-editing efficiency in brain tissues. The precise molecular association of GLP2 peptides within the AELN/RNP formulation remains to be elucidated.

While GLP2R expression has been reported in neuronal tissues, further studies are required to define the precise cellular targets and molecular mechanisms underlying this enhanced delivery.^31^ Importantly, the present study does not address the molecular mechanism of RNP protection or encapsulation but rather demonstrates that association with AELNs is sufficient to preserve functional genome-editing activity under serum and *in vivo* conditions. These findings demonstrate the value of GLP2 modification for enhancing the targeting capability of CRISPR/Cas9 delivery systems.

From a translational perspective, scalability and reproducibility are critical considerations for clinical development. While large-scale production was not the primary focus of the present study, the AELN platform is inherently compatible with scalable manufacturing. Acerola-derived nanovesicles can be produced from a stable, abundant raw material, and tangential flow filtration (TFF) enables high-throughput isolation with controllable yield and particle characteristics.

Importantly, Cas9 is a recombinant protein for which large-scale production and quality-control frameworks are already well established in other therapeutic contexts. The non-covalent assembly of AELNs with RNP complexes further supports batch-to-batch reproducibility and process standardization. Although additional optimization and formal validation will be required for clinical-grade manufacturing, these features indicate that the AELN-RNP system is well positioned for future scale-up and translational development.

This study has several limitations. While intranasal administration showed high efficiency, oral administration under the same dosing conditions did not result in detectable brain delivery or genome-editing activity in the target regions (Figure S6). The present study does not aim to quantitatively evaluate whole-brain biodistribution or systemic transport efficiency of AELN/RNP complexes; rather, our conclusions are limited to the experimental observations that intranasally administered AELNs enable detectable delivery of Cas9 to periventricular brain regions and support genome-editing activity at the target locus. Although AELNs exhibit relative resistance to certain digestive enzymes,^17^ oral administration under the conditions tested did not result in detectable brain delivery or genome-editing activity. While intestinal uptake of AELNs has been confirmed *in vitro* using epithelial transport models, efficient brain delivery following oral administration likely remains limited by post-absorption processes, including systemic stability, nonspecific clearance, and transport across the BBB.^32^ Addressing these barriers will require further advancements in protective coatings,^33^ bioencapsulation, formulation technologies, or stabilization methods to enhance the resilience and bioavailability of AELN/RNP complexes. In addition, future optimization will focus on modifying AELN properties that are directly relevant to brain accessibility, such as reducing particle size and enhancing targeting to brain-associated receptors, with the specific aim of improving BBB traversal rather than gastrointestinal stability alone.

Another limitation is the lack of long-term safety and off-target effect assessments. This study primarily focused on short-term efficacy, and further investigations into the prolonged effects and specificity of AELN-mediated delivery systems are crucial for clinical translation. In future studies, comprehensive safety evaluation will be essential, including long-term observation following repeated administration, assessment of innate and adaptive immune responses, and unbiased genome-wide off-target analyses using next-generation-sequencing-based approaches. Importantly, the use of CRISPR/Cas9 RNP delivery, rather than DNA- or mRNA-based expression systems, is expected to limit the duration of Cas9 activity *in vivo* and may reduce off-target risks, although this must be rigorously validated. In addition, evaluating inflammatory responses and tissue integrity in the central nervous system will be critical to support the translational development of AELN-based genome-editing therapeutics. Additionally, scaling up the production of AELN/RNP complexes for clinical trials remains a significant technical challenge.

However, despite these challenges, the findings of this study represent a significant advancement in the development of noninvasive gene-editing therapies. The successful targeting of *C9orf72* mutations in ALS models demonstrates the therapeutic potential of AELNs for treating neurodegenerative diseases. Notably, no overt acute immune responses were observed following administration of the AELN/RNP complex, as assessed by body-weight monitoring and serum cytokine measurements (Figure S7); however, comprehensive immunogenicity profiling will be required in future studies. Future directions should include testing this approach in other central nervous system disorders, such as Alzheimer and Parkinson diseases, by exploring alternative ligands to enhance targeting. Moreover, optimizing oral delivery systems could provide a noninvasive, patient-friendly treatment option, further broadening the accessibility of gene therapy.^34^

In conclusion, the AELN-based CRISPR/Cas9 delivery system offers a promising and efficient approach for noninvasive gene therapy. By leveraging the unique properties of plant-derived exosomes, this study provides a foundation for further research into safe and scalable delivery systems for genetic editing tools. Continued advancements in this field will transform the treatment landscape for neurodegenerative and genetic disorders.

## Materials and methods

### Ethics

All animal experiments were conducted in compliance with the institutional guidelines of the Animal Experimental Center of Tokyo Medical University/Animal Biosafety Level-II Laboratory for Use of Animals. The experimental protocols were approved by the Institutional Animal Care and Use Committee of Tokyo Medical University (approval number R5-011).

### Cell culture

The human embryonic kidney cell line HEK293 was purchased from the American Type Culture Collection (ATCC; Manassas, VA, USA). HUVECs were purchased from LONZA Inc. (Basel, Switzerland), and 3LL cells were obtained from JCRB (JCRB1349; Osaka, Japan). These cell lines were purchased as authenticated stocks and accompanied by the supplier’s short tandem repeat (STR) profiling data. Cells were used within five passages from thawing and for a limited experimental period to minimize the risk of cross-contamination or genetic drift; therefore, no additional STR analysis was performed in our laboratory. All cell lines were confirmed to be mycoplasma-free via PCR-based testing prior to use. HEK293 and 3LL cells were maintained using Dulbecco’s modified Eagle medium (DMEM; Gibco, Invitrogen, Carlsbad, CA, USA) supplemented with 10% heat-inactivated fetal bovine serum (HyClone, Thermo Fisher Scientific, Waltham, MA, USA). HUVECs were maintained using EBM-2 basal medium and EGM-2 SingleQuots Supplements (Lonza). All cells were maintained at 37°C in a humidified atmosphere containing 5% CO_2_, and the adherent cells were harvested through trypsinization.

### Extraction and characterization of AELNs

Using the exoEasy Maxi Kit (QIAGEN, Hilden, Germany), 8 mL of acerola cherry juice was passed through a 0.45-μm polyvinylidene fluoride filter (Millipore, Billerica, MA, USA). The filtered juice was then mixed with an equal volume of buffer XBP and transferred to an exoEasy spin column. All processes were performed according to the manufacturers’ protocols. Subsequently, 800 μL buffer XE was added, and the mixture centrifuged at 5,000 × g for 5 min. The flowthrough was then concentrated via ultracentrifugation at 100,000 × g for 70 min at 4°C using a TLA-110 rotor (Beckman Coulter, Brea, CA, USA) and buffer XE replaced with 30 μL phosphate-buffered saline (PBS) (Figure 1A).

### Measurement of the number of AELNs

NTA was performed using the NanoSight LM10 system (Malvern Panalytical, Herrenberg, Germany). AELNs were illuminated with a 450-nm blue laser, and their Brownian motion was recorded in 90-s videos, which were analyzed using NTA 2.0 (Malvern Panalytical). Samples were diluted with PBS to achieve a particle concentration suitable for NTA analysis (1 × 10^8^–2.5 × 10^9^ particles/mL). Capture, shutter, and gain settings for analysis were manually adjusted according to the manufacturer’s instructions and kept consistent throughout each experiment. Multiple video recordings of each sample were averaged to determine the total concentration of nanoparticles (Figure 1B).

### Observation of the morphology of AELNs

For TEM analysis, AELNs were fixed with 4% paraformaldehyde and 4% glutaraldehyde in 0.1 M phosphate buffer (pH 7.4) at room temperature (∼25°C) and then cooled to 4°C. The samples were adsorbed onto carbon-coated 400-mesh grids and stained with 2% phosphotungstic acid solution (pH 7.0) for 30 s. Observations were performed using a JEM-1200EX transmission electron microscope (JEOL, Tokyo, Japan) at 80 kV (Figure 1C).

### gRNA preparation

To target specific genomic sequences with gRNAs, CRISPR RNAs (crRNAs) were designed and synthesized as follows. For the human *GLA* gene, a gRNA targeting the 5′-ATTGGCAAGGACGCCTACCATGG-3′ sequence was purchased from Integrated DNA Technologies (IDT; Coralville, IA, USA). For the *C9orf72* gene, gRNAs targeting sequences before and after the GGGGCC repeat in humans and mice were designed. For the human *C9orf72* gene, gRNAs corresponding to the sequence before (gRNA-1, 5′-TGTAGCAAGCTCTGGAAC-3′) and after the repeat sequence (gRNA-2, 5′-GAAGAGGCGCGGGTAGAAG-3′) were purchased from IDT based on the protocol described in a previous study^19^ (Figure 3A). Similarly, for the mouse *C9orf72* gene, gRNA-1m (5′-CTCGCAACTTAACTCCACAA-3′) and gRNA-2m (5′-TTGGCCTTGCAGGAGTTGCG-3′) were purchased from IDT (Figure 4A).

### AELN/RNP complex formation

The gRNAs targeting specific genomic sequences were prepared by adding 1 μL Alt-R CRISPR-Cas9 crRNA (100 μM; IDT) and 1 μL Alt-R CRISPR-Cas9 tracrRNA (100 μM; IDT) to 98 μL nuclease-free duplexing buffer (IDT). The mixture was incubated at 95°C for 5 min, followed by a further 20 min at room temperature.

For Cas9 protein preparation, 1 μL of Alt-R *S. pyogenes* Cas9 Nuclease V3 or Alt-R *S. pyogenes* HiFi Cas9 Nuclease V3 (62 μM; IDT) was added to 61 μL Opti-MEM I Reduced Serum Media (Thermo Fisher Scientific).

When targeting the human *GLA* gene, gRNA and Cas9 protein were mixed at an equimolar ratio, resulting in an RNP solution containing 30 pmol Cas9 per reaction. To target the C9orf72 genes in humans and mice, two gRNAs (gRNA-1 and gRNA-2) and Cas9 protein were mixed at an equimolar ratio, yielding an RNP solution containing 30 pmol Cas9 per reaction. The mixtures were then incubated at room temperature for 10 min to allow RNP complex formation.

To assemble the AELN/RNP complex, AELNs quantified by NTA were used at 4 × 10^8^ particles per reaction (corresponding to approximately 10 μL of the AELN preparation). The RNP solution (corresponding to 30 pmol Cas9) was mixed with AELNs, resulting in an approximate ratio of 1.3 × 10^7^ AELN particles per pmol Cas9. The mixture was incubated on ice for 20 min to allow complex formation (Figure 2A).

For RNAiMAX (Thermo Fisher Scientific) controls, RNPs corresponding to 30 pmol Cas9 were complexed with RNAiMAX according to the manufacturer’s protocol. Additionally, human-breast-milk-derived exosomes (Cosmo Bio, Tokyo, Japan) were used as another control for AELNs. For this control, exosomes were prepared at a concentration of 2 × 10^8^ particles per well (corresponding to the same particle-to-cell ratio as AELNs). All concentrations and particle numbers were kept constant across experiments unless otherwise specified.

### Confirmation of AELN and RNP binding using FRET analysis

To confirm the binding of AELNs and RNPs, FRET analysis was performed. The analysis utilized Alt-R S.p. Cas9-GFP V3 (IDT) and AELNs labeled with PKH26 (Sigma-Aldrich, St. Louis, MO, USA). AELNs were labeled according to the manufacturer’s instructions.

For FRET analysis, the previously prepared RNP complex was modified by replacing the Cas9 protein with Alt-R S.p. Cas9-GFP V3. Subsequently, PKH26-labeled AELNs were added to the RNP complex and fluorescence measurement initiated immediately after mixing using a fluorescence plate reader (Infinite F200 PRO; Tecan Life Sciences, Männedorf, Switzerland).

Fluorescence signals were measured every 4 min using a fluorescence plate reader. FRET signals were recorded using a filter set with excitation and emission wavelengths of 488 and 580 nm, respectively (FRET filter; Tecan Life Sciences). Temporal monitoring of FRET fluorescence intensity was conducted over 30 min to evaluate the kinetics and stability of AELN–RNP complex formation.

### Visualization of the cell transduction of AELN/RNP complexes

HEK293 cells were seeded at a density of 2.0 × 10^4^ cells per chamber on eight-chamber slides (Thermo Fisher Scientific) in 500 μL of DMEM and cultured for 24 h at 37°C. AELN/RNP complexes were prepared as described above, except that Alt-R S.p. Cas9-GFP V3 (62 μM) was used instead of Alt-R S.p. Cas9 Nuclease V3. Cells were treated with AELN/RNP complexes at a dose of 4 × 10^4^ AELN particles per cell. Cells treated with RNPs alone served as controls.

After incubation at 37°C for 24 h, cells were washed with PBS and stained with Hoechst 33342 to visualize nuclei and with an endosomal marker as indicated. Fluorescence images were acquired using identical microscope settings across all conditions. Images were analyzed using ImageJ software to quantify cellular uptake, as described in the figure legends.

### *In vitro* transfection of the AELN/RNP complex

The genome-editing efficiency of the AELN/RNP complex was evaluated in HEK293 cells, HUVECs, and 3LL cells. Cells were seeded at a density of 1.0 × 10^4^ cells per well in 100 μL DMEM in 96-well plates (Corning Inc., Corning, NY, USA) and cultured for 24 h prior to transfection.

AELN/RNP complexes were directly added to the culture medium at a constant dose of 4 × 10^4^ AELN particles per cell unless otherwise specified. After 24 h of incubation, cells were harvested and genomic DNA extracted for further analysis (Figure 2A).

Commercially available human-breast-milk-derived exosomes were purchased from Funakoshi Co., Ltd. (Tokyo, Japan). According to the manufacturer, these exosomes were isolated from donor milk by differential ultracentrifugation and characterized by NTA, TEM, and exosomal marker profiling (CD63, CD81). Exosomes were resuspended in PBS and stored at −80°C until use.

### Serum stability assay

To evaluate the stability and functional integrity of CRISPR/Cas9 ribonucleoprotein (RNP) complexes in serum, pre-formed AELN/RNP complexes were incubated in the presence of serum prior to cell treatment. AELN/RNP complexes were prepared as described above at a particle-to-protein molar ratio of 10:1 by mixing AELNs (quantified by NTA) with Cas9 RNPs on ice for 20 min.

The complexes were incubated with either 10% (v/v) human serum or mouse serum at 37°C for the indicated time points (0, 5, 30, 60, and 120 min). Following serum incubation, the samples were diluted 10-fold with complete culture medium to minimize serum-associated cytotoxicity and immediately applied to HEK293 cells under standard culture conditions.

Genome-editing activity was assessed 24 h after treatment by Sanger sequencing of the target locus, and indel frequencies were quantified using Tracking of InDels by Decomposition (TIDE) analysis. As a control, RNPs without AELNs (RNP only) were subjected to the same serum incubation and dilution procedures prior to cell treatment.

Unless otherwise stated, all AELN/RNP complexes used in *in vitro* experiments, including the serum stability assay, were prepared using the same particle-to-protein molar ratio (10:1).

### Targeted drug delivery using GLP2 peptides

In this study, GLP2 peptides were introduced during the preparation of AELN/RNP formulations by simple co-incubation, without chemical conjugation or intentional covalent modification of either AELNs or RNP components. Initially, a HEK293 cell line stably expressing GLP2R (HEK293/GLP2R) was developed. Wild-type HEK293 cells were seeded in a 10-cm cell culture dish (Falcon, Corning, NY, USA) and cultured in 10 mL DMEM for 24 h. The p2LP-GLP2R plasmid, which encodes the human GLP2R (NM_004246.4) under a CAG promoter, was purchased from VectorBuilder (Chicago, IL, USA) and introduced into the cells, following the manufacturer’s instructions. After incubation at 37°C for 24 h, the medium was replaced, and the cells further cultured for another 24 h. Subsequently, limiting dilution cloning was performed to isolate single-cell clones, and a stable HEK293/GLP2R cell line was established. RNA was extracted from these cells to confirm GLP2R expression.

RNA extraction was performed using the miRNeasy Mini Kit (Qiagen), followed by cDNA synthesis, according to the manufacturer’s protocol. Quantitative RT-PCR was conducted using Assay-on-Demand primers and TaqMan Universal PCR Master Mix Reagent (Applied Biosystems, Waltham, MA, USA) to verify the successful generation of HEK293/GLP2R cells. β-Actin (ACTB) was used as an internal normalization control.

For experiments using HEK293/GLP2R cells, GLP2-modified AELN/RNP complexes were prepared by incubating AELN/RNP complexes (4 × 10^8^ AELN particles per reaction) with GLP2 peptide at a final amount of 0.2 μg in Opti-MEM I Reduced Serum Medium. The mixture was incubated at room temperature for 20 min before being applied to the cells.

For *in vivo* experiments, GLP2-modified AELN/RNP complexes were prepared using the same protocol as described above, by incubating AELN/RNP complexes (4 × 10^8^ particles per dose) with 0.2 μg of GLP2 peptide in Opti-MEM I Reduced Serum Medium for 20 min. The prepared complexes were then administered intranasally.

The same GLP2 modification conditions were used throughout all *in vitro* and *in vivo* experiments unless otherwise specified.

### Competitive uptake assay for GLP2-peptide-modified AELNs

The GLP2 peptide used in this study (amino acid sequence: HADGSFSDEMNTILDNLAARDFINWLIQTKITD) was synthesized at >95% purity by Cosmo BIO Co., Ltd. (catalog no. PP24020852). The lyophilized peptide was reconstituted in sterile PBS (pH 7.4) to a stock concentration of 10 μg/μL and stored at −20°C until use.

For GLP2 modification, PKH26-labeled AELNs quantified by NTA were incubated with GLP2 peptide at a final amount of 0.2 μg per 4 × 10^8^ AELN particles in Opti-MEM I Reduced Serum Medium. The mixture was incubated at room temperature for 20 min to allow noncovalent peptide association with AELNs. Unmodified AELNs were processed in parallel without GLP2 peptide.

Wild-type HEK293 cells were left unlabeled, whereas GLP2R-overexpressing HEK293 cells were pre-labeled with PKH67 (Sigma-Aldrich) according to the manufacturer’s protocol. The two cell populations were then co-cultured at a 1:1 ratio on glass-bottom dishes. Cells were incubated with PKH26-labeled AELNs (modified or unmodified) at a final dose corresponding to 4 × 10^4^ AELN particles per cell (4.0 × 10^8^ particles/mL) for 4 h at 37°C.

After incubation, cells were washed with PBS, nuclei were counterstained with Hoechst 33342, and fluorescence images were acquired under identical settings for all samples. PKH26 fluorescence intensity per cell was quantified using ImageJ.

### Intranasal administration of the AELN/RNP complex in mice

To evaluate *in vivo* genome-editing and brain delivery of the AELN/RNP complex, C57BL/6 male mice (8 weeks old; *n* = 5 per group) were used.

AELN/RNP complexes were prepared as described above. For GLP2-modified formulations, AELNs quantified by NTA were incubated with GLP2 peptide at a final amount of 0.2 μg per 4 × 10^8^ AELN particles in Opti-MEM I Reduced Serum Medium for 20 min at room temperature prior to RNP complex formation.

For each mouse, a total of 2.0 × 10^10^ AELN particles, 300 pmol Cas9 protein complexed with the corresponding gRNAs, and 10 μg GLP2 peptide were administered intranasally in a total volume of 5 μL (2.5 μL per nostril) using an ultrafine pipette tip under light anesthesia. Vehicle-treated control mice received an equal volume of sterile PBS (pH 7.4) using the same protocol. At 24 h post-administration, mice were euthanized, and brain tissues were harvested for genomic DNA extraction and downstream analyses.

### *In vivo* tracking of intranasally delivered AELNs

For *in vivo* tracking experiments, AELNs were labeled with PKH26 (Sigma-Aldrich) according to the manufacturer’s instructions. Excess unincorporated dye was removed by ultracentrifugation at 100,000 × g for 70 min at 4°C. PKH26-labeled AELNs were quantified by NTA and administered intranasally to C57BL/6 male mice (8 weeks old; *n* = 5) at a dose of 2.0 × 10^10^ particles in a total volume of 5 μL (2.5 μL per nostril) under light anesthesia.

At 24 h after administration, mice were perfused with PBS, and brains were harvested, embedded in OCT, and cryosectioned at 10 μm.

Brain sections were counterstained with Hoechst 33342 to visualize nuclei and imaged using a fluorescence microscope (Nikon) under identical acquisition settings for all samples.

For semiquantitative analysis, mean PKH26 fluorescence intensity within predefined periventricular regions of interest (ROIs; width 3 μm) was measured using ImageJ and normalized to adjacent background ROIs. Values were averaged per animal and used for group comparisons.

To assess acute tolerability and potential immune activation, mice were administered the AELN/RNP complex at the same dose used in the *in vivo* experiments. Body weight was monitored on days 0, 1, 3, and 7 after administration. For cytokine analysis, blood samples were collected on days 1 and 3, and serum levels of interleukin (IL)-6 and interferon gamma (IFN-γ) were measured by ELISA using commercially available kits (LBIS Mouse IL-6 ELISA Kit and LBIS Mouse IFN-γ ELISA Kit; FUJIFILM Wako Pure Chemical Corporation, Japan) according to the manufacturer’s instructions.

### Genomic DNA extraction and Sanger sequencing

Genomic DNA was extracted from cultured cells (*in vitro* experiments) and brain tissues (*in vivo* experiments) using the DNeasy Blood and Tissue Kit (Qiagen, Hilden, Germany), following the manufacturer’s protocol. The extracted genomic DNA was subjected to PCR amplification using KOD FX (TOYOBO, Osaka, Japan). For targeting the *GLA* gene, the following forward (5′-GGTTAGCGGAACGTCTTACG-3′) and reverse primers (5′-TTATCCCCAGCAAACTGTCC-3′) were used. For targeting the *C9orf72* gene in HEK293 cells and HUVECs, the following forward (5′-GGGTCAAGCAAGAGCAGGTG-3′) and reverse primers (5′-ACAAGGGATGGGGATCTG-3′) were used. For targeting the *C9orf72* gene in 3LL cells and mouse brain tissue, the following forward (5′-ACGTGAAAGATGGCGTTTGT-3′) and reverse primers (5′-GGATGGAGTAAGGACGGACA-3′) were used. All primers were synthesized by Eurofins Genomics (Tokyo, Japan).

PCR reactions were prepared according to the manufacturer’s instructions. The PCR conditions consisted of an initial pre-denaturation at 94°C for 2 min, followed by 30 cycles of denaturation at 98°C for 10 s, annealing at 56°C for 30 s, and extension at 68°C for 30 s. The PCR products were analyzed via gel electrophoresis and DNA bands of interest subsequently excised. DNA was extracted from the gel slices using the QIAquick Gel Extraction Kit (Qiagen), following the manufacturer’s protocol.

Sanger sequencing was performed by Azenta Life Sciences (Tokyo, Japan). Genome-editing efficiency, specifically indel formation, was analyzed using TIDE, an online tool available at https://tide.nki.nl. For population-level quantification of genome editing at the C9orf72 locus, PCR products amplified with primers upstream of gRNA-1 and downstream of gRNA-2 were directly sequenced by Sanger without gel purification. Indel frequencies at each cut site were quantified by Tracking of Indels by Decomposition (TIDE) analysis using the corresponding untreated control sample as reference.

### Statistical analysis

Data were analyzed using Student’s *t* test or ANOVA, as appropriate, to compare the treated and control groups. Prior to selecting the statistical test, data distribution was assessed using both the Shapiro-Wilk and the D’Agostino-Pearson omnibus tests to evaluate normality. Parametric tests (Student’s *t* test or ANOVA) were applied to datasets meeting the criteria for normal distribution in both tests, and nonparametric alternatives were used otherwise. A *p* value <0.05 was considered statistically significant. Data are presented as mean ± standard deviation (SD). All statistical analyses were performed using GraphPad Prism v.10.5.0 (GraphPad Software, San Diego, CA, USA).

## Data and code availability

The datasets generated and/or analyzed during the current study are not publicly available due to ongoing patent applications and intellectual property considerations; however, they are available from the corresponding author upon reasonable request.

## Acknowledgments

This work was supported by the 10.13039/100009619Japan Agency for Medical Research and Development (AMED) under grant number JP25ym0126183h0001. We thank members of the Kuroda laboratory for helpful discussions and technical assistance. We also thank the staff of the Animal Experimental Center at Tokyo Medical University for support with the *in vivo* experiments.

## Author contributions

Y.N. contributed to data collection and manuscript writing. T.U. contributed to research design and planning, data collection, data analysis, and manuscript writing. T.H. contributed to the research design and planning, data collection, data analysis, and technical expertise. T.N., S.O., and Y.H. contributed to data collection. M.K. contributed to the research design and planning, as well as data analysis. T.O. contributed to technical expertise and manuscript writing. M.K. contributed to the research design and planning, data analysis, technical expertise, manuscript writing, and funding acquisition.

## Declaration of interests

The authors declare no competing interests.

## Supplemental information

Document S1. Figures S1–S7

Document S2. Article plus supplemental information
