# Supplementary material for: Exosome-like nanovesicles from acerola for CRISPR-Cas9 ribonucleoprotein delivery to the central nervous system
Source: Mol Ther Nucleic Acids. 2026 Mar 12;37(2):102896. doi: 10.1016/j.omtn.2026.102896 (PMC13022685; doi:10.1016/j.omtn.2026.102896)
Supplement: Document S1. Figures S1–S7 [file mmc1.pdf]

OMTN, Volume *37*

Supplemental information

Exosome-like nanovesicles from acerola for CRISPR-Cas9 ribonucleoprotein delivery to the central nervous system

Yui Nagamatsu, Tomohiro Umezu, Taehun Hong, Takahide Niijima, Shin-ichiro Ohno, Yuichiro Harada, Kohsuke Kanekura, Takahiro Ochiya, and Masahiko Kuroda

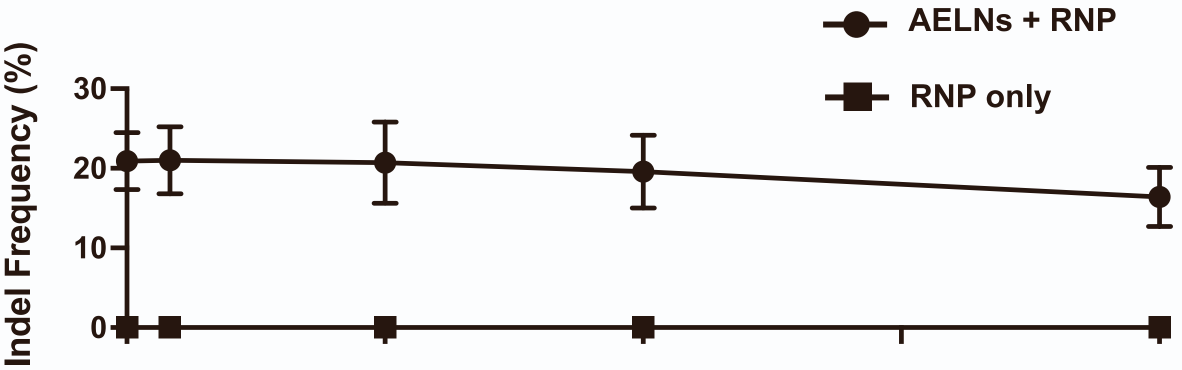

Figure S1. Time-course serum challenge assay of AELN/RNP complexes in mouse serum.

AELN/RNP complexes (particle-to-protein molar ratio of 10:1) or RNPs alone were incubated in 10% mouse serum at 37 ºC for the indicated times (0, 5, 30, 60, and 120 min). Following serum incubation, samples were diluted in complete culture medium and applied to HEK293 cells. Genome-editing efficiency was quantified by Tracking of InDels by Decomposition (TIDE) analysis 24 h after cellular treatment. Data are presented as mean ± SD from n = 3 independent experiments. Time 0 min indicates no serum incubation.

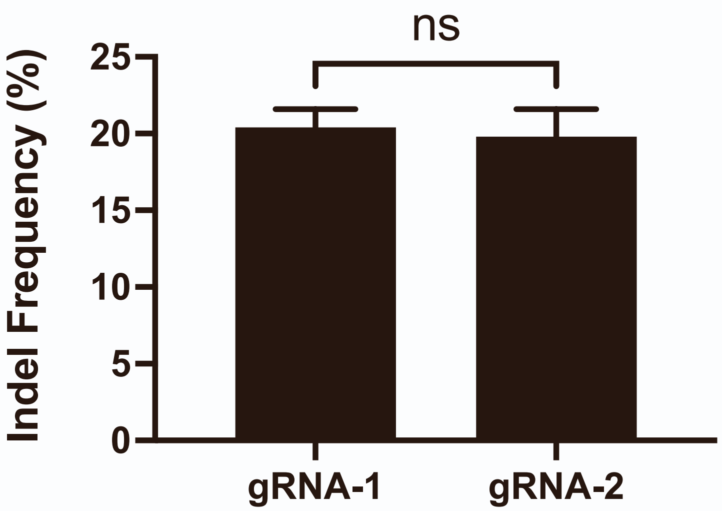

Figure S2. Comparison of genome-editing efficiencies of gRNA-1 and gRNA-2 targeting the C9orf72 repeat region.

Indel frequencies induced by gRNA-1 and gRNA-2 in HEK293 cells were quantified by TIDE analysis. Data are presented as mean ± SD from n = 3 independent experiments. No statistically significant difference was observed (ns; $P > 0.05$).

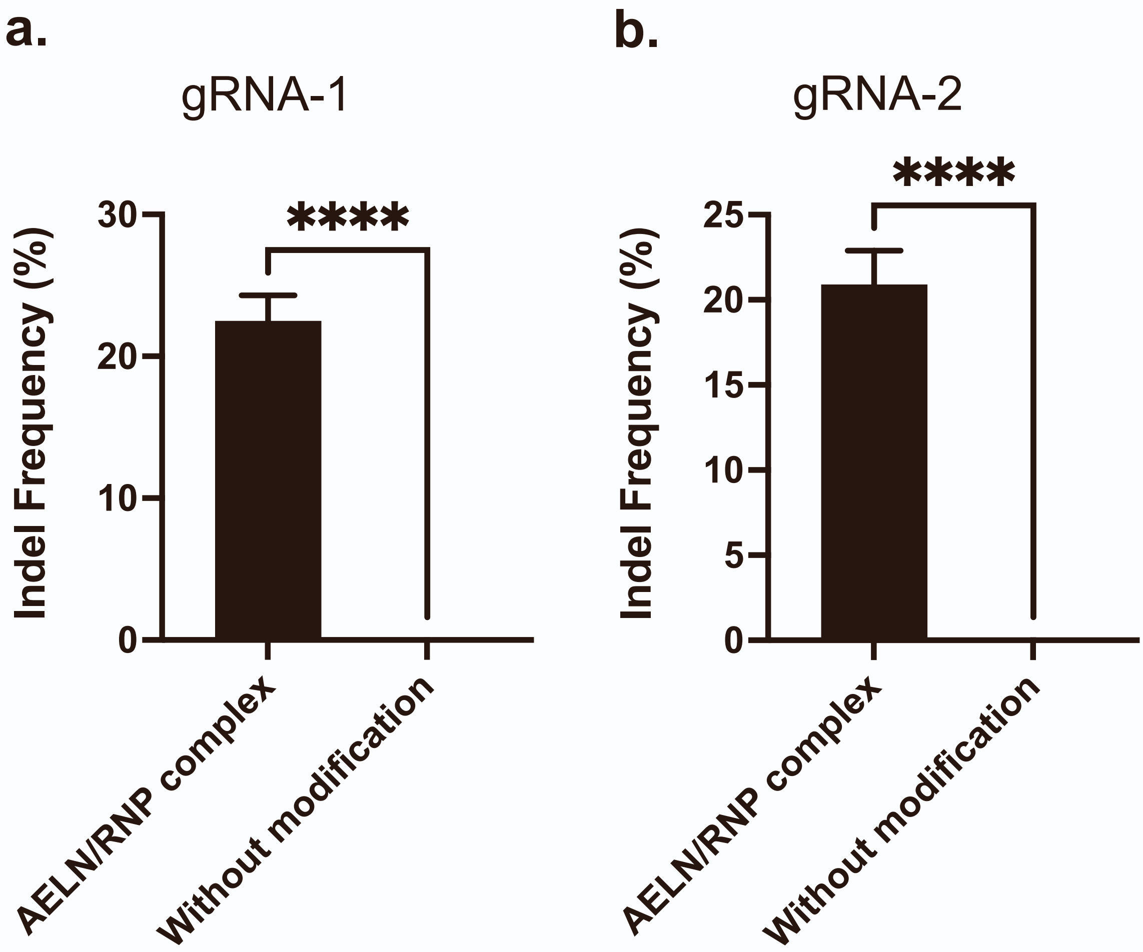

Figure S3. Population-level quantification of indel formation at the C9orf72 locus using TIDE.

PCR products amplified with primers located upstream of gRNA-1 and downstream of gRNA-2 (as in Fig. 3b–c) were subjected to Sanger sequencing without gel purification. Indel frequencies at the gRNA-1 (a) and gRNA-2 (b) cut sites were quantified by TIDE using "without modification" as the reference control. Data are shown as mean ± SD from n = 3 independent AELN/RNP complex preparations; individual data points are overlaid. Statistical significance was assessed using an unpaired two-tailed Student's t-test. ****$p < 0.0001$.

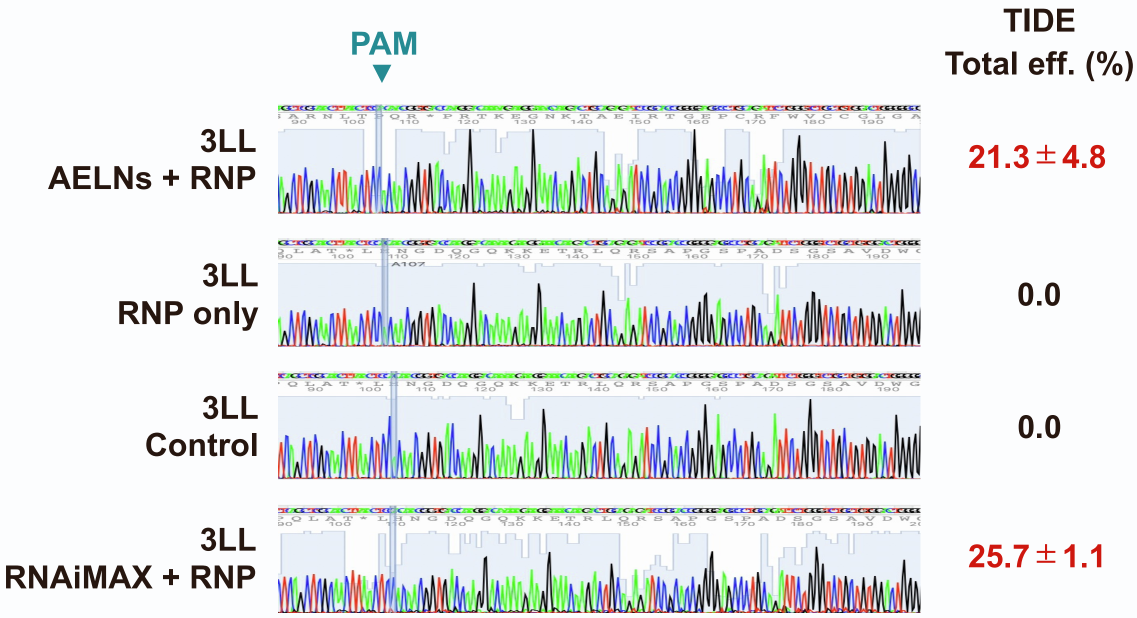

Figure S4. Sanger sequencing chromatograms of the murine C9orf72 locus in 3LL cells.

PCR products amplified from 3LL mouse cells treated with AELN + RNP, RNP only, RNAiMAX (Lipofectamin) + RNP, or untreated control were subjected to Sanger sequencing at the C9orf72 target locus. Chromatograms are shown for the region downstream of the gRNA cut site. In addition to base-call peaks, the confidence trace (gray background) is displayed, reflecting the signal consistency at each nucleotide position. Reduced confidence and characteristic downstream peak dispersion are observed specifically in genome-edited samples, consistent with mixed sequencing populations caused by indel formation. Quantitative genome-editing efficiencies for these samples are provided in the main Figure 3e based on TIDE analysis of the same sequencing data. PAM, protospacer adjacent motif.

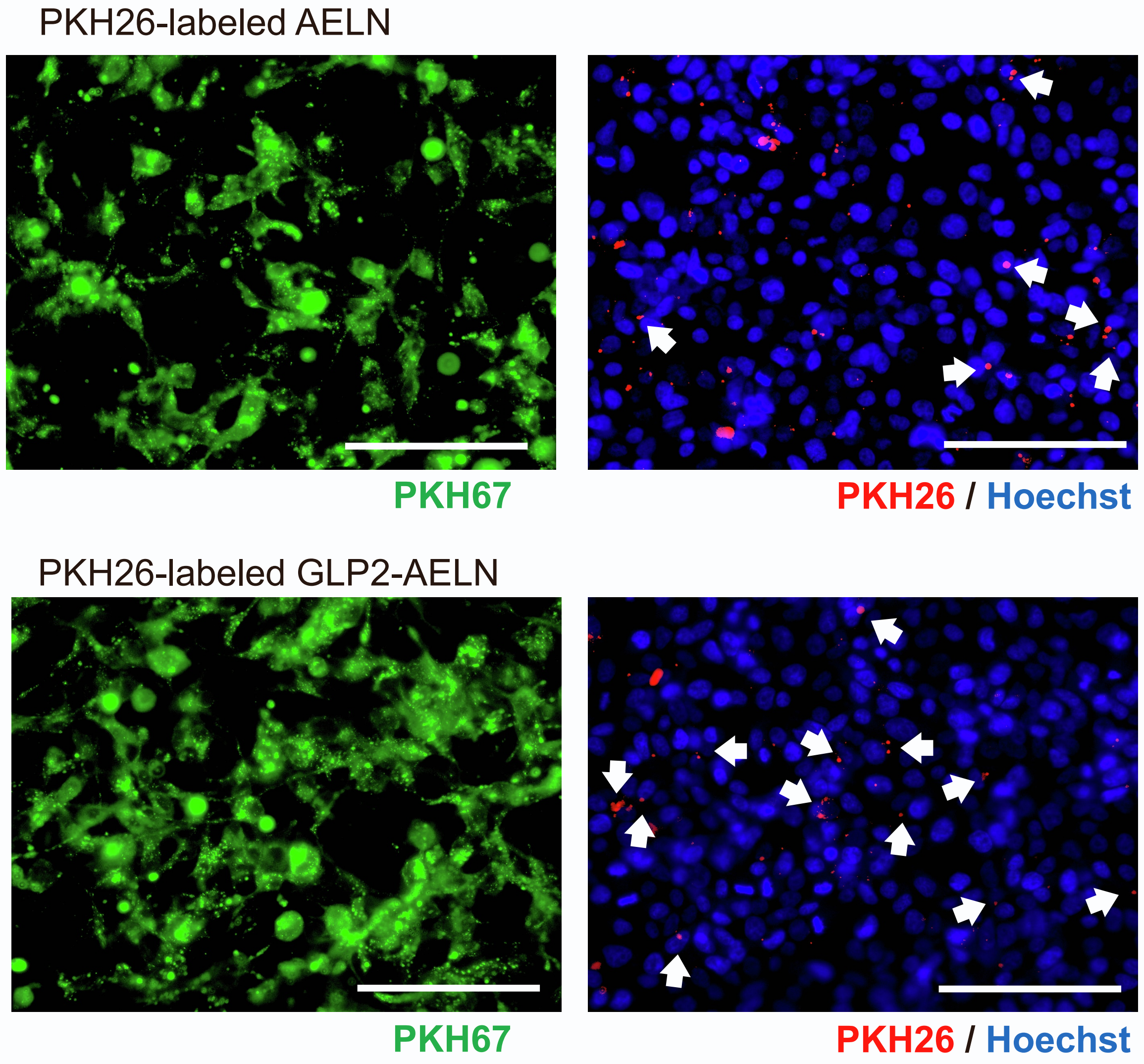

Figure S5. Channel-separated fluorescence images supporting receptor-dependent uptake of GLP2-modified AELNs.

Channel-separated fluorescence images corresponding to the competitive co-culture assay shown in Fig. 4c–d. Wild-type and GLP2R-overexpressing HEK293 cells were co-cultured at a 1:1 ratio, with GLP2R-overexpressing cells pre-labeled with PKH67 (green). PKH26-labeled unmodified AELNs or GLP2-modified AELNs (GLP2–AELNs) were added to the cultures.

Panels show 20× magnified views of red-channel (PKH26) and green-channel (PKH67) images for the same fields. Arrows indicate corresponding PKH26-positive puncta and PKH67-positive cells, facilitating identification of vesicle localization within GLP2R-expressing cells across channels. Red-channel images demonstrate comparable uptake of unmodified AELNs by both cell types, whereas GLP2–AELNs show preferential uptake by PKH67-positive (GLP2R-overexpressing) cells. These channel-separated images clarify receptor-dependent uptake that may be less visually apparent in merged images due to color overlap. Images are representative of n = 3 independent experiments. Scale bar: 500 μm.

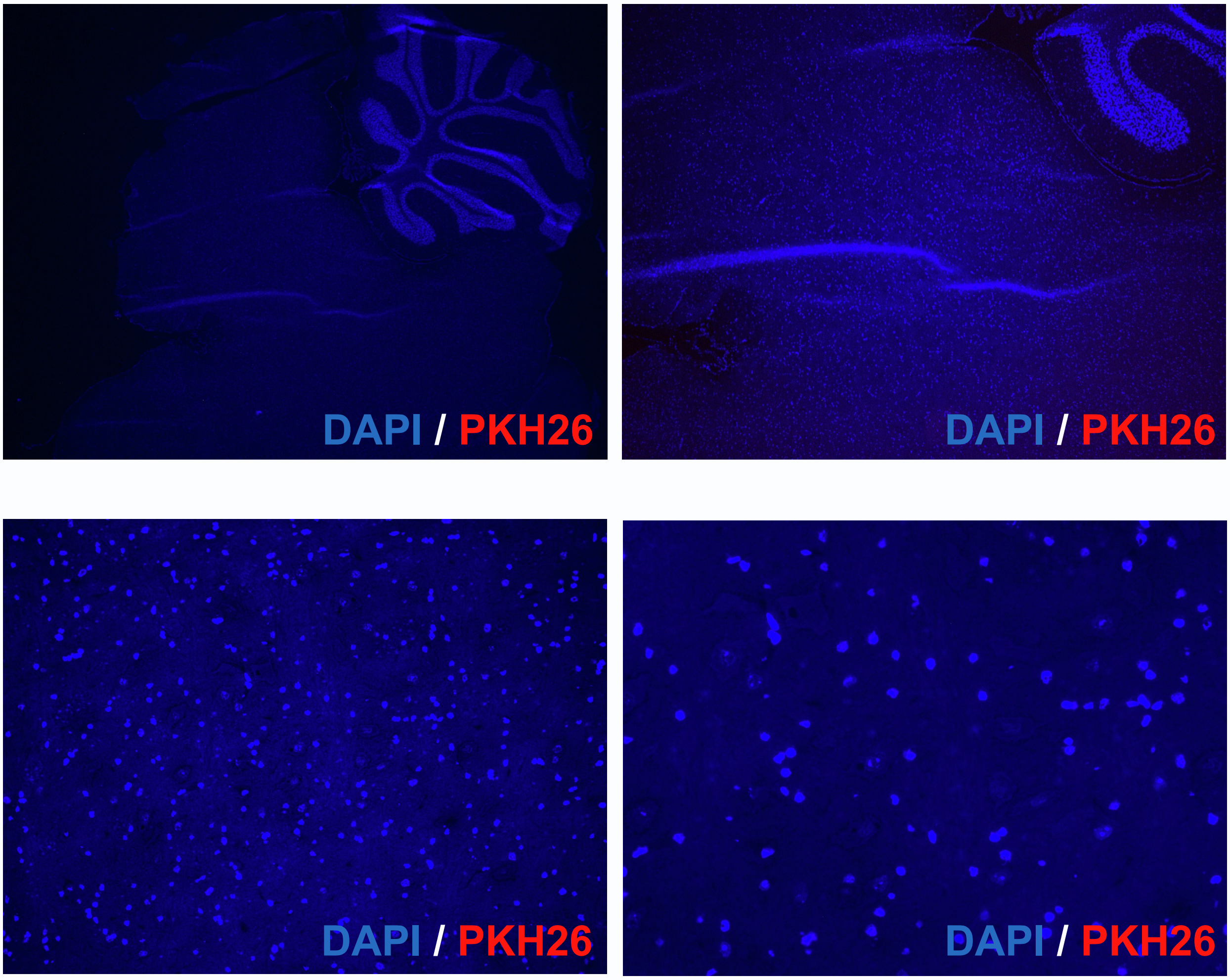

Figure S6. Evaluation of brain delivery following oral administration of PKH26-labeled AELNs.
Cryosections of mouse brains collected 24 h after oral administration were counterstained with DAPI. No detectable PKH26 fluorescence was observed in brain sections under the same dosing conditions used for intranasal administration, indicating the absence of detectable brain delivery via the oral route.

a.

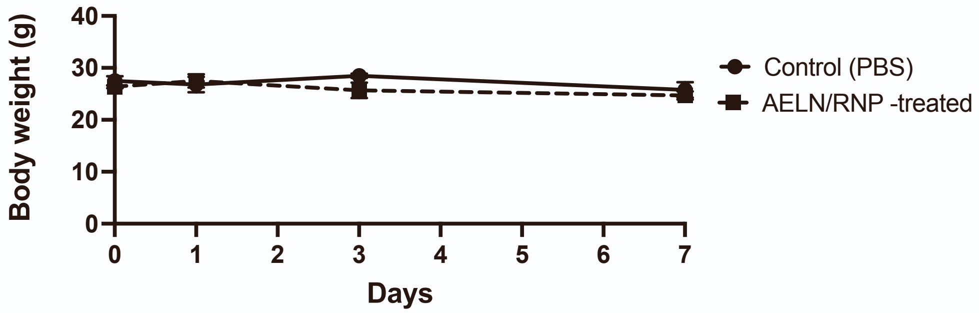

b.

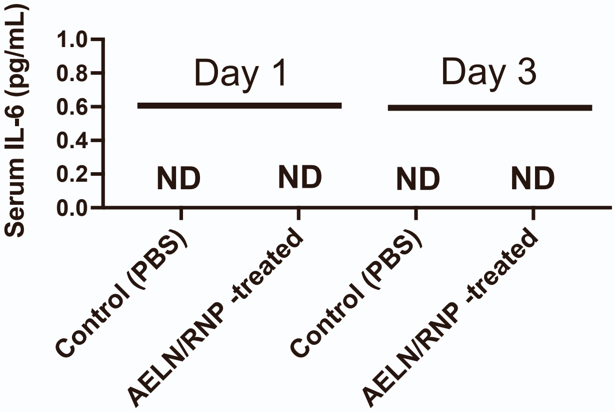

c.

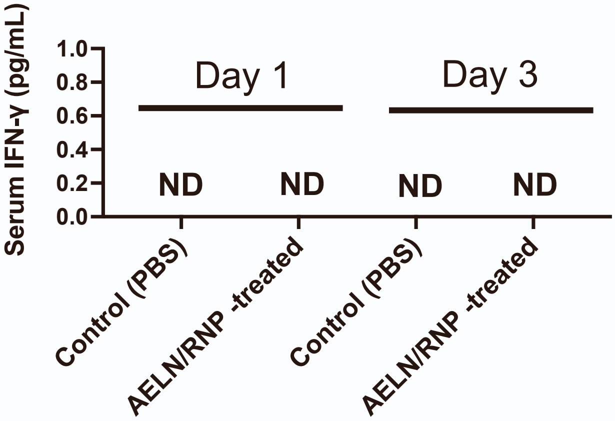

Figure S7. In vivo tolerability and acute immunogenicity assessment of the AELN/RNP complex.

(a) Body-weight changes in mice following administration of the AELN/RNP complex at the same dose used in the in vivo experiments. Body weight was monitored on days 0, 1, 3, and 7 after administration. No apparent body-weight loss or abnormal trend was observed compared with the control group (mean ± SEM).

(b) Serum IL-6 levels measured on days 1 and 3 after administration. All measurements were below the assay detection limit in both the control and AELN/RNP-treated groups and are shown as ND.

(c) Serum IFN-γ levels measured on days 1 and 3 after administration. All measurements were below the assay detection limit in both groups and are shown as ND.

These results indicate the absence of overt acute or subacute immune responses under the tested conditions.
